# Cerebral Vasoreactivity Evaluated by Transcranial Color Doppler and Breath-Holding Test in Patients after SARS-CoV-2 Infection

**DOI:** 10.3390/jpm11050379

**Published:** 2021-05-06

**Authors:** Marino Marcic, Ljiljana Marcic, Barbara Marcic, Vesna Capkun, Katarina Vukojevic

**Affiliations:** 1 Department of Neurology, University Hospital Center Split, 21000 Split, Croatia; 2 Department of Radiology, Polyclinic Medicol, 2100 Split, Croatia; lmarcic@mefst.hr; 3 Department of Medical Genetics, University of Mostar School of Medicine, 88000 Mostar, Bosnia and Herzegovina; barbara.marcic@mef.sum.ba; 4 Department of Anatomy, Histology and Embryology, University of Split School of Medicine, 21000 Split, Croatia; vesna.capkun@mefst.hr

**Keywords:** SARS-CoV-2, nonspecific neurological symptoms, transcranial color Doppler, vasoreactivity

## Abstract

From the beginning of the SARS-CoV-2 virus pandemic, it was clear that the virus is highly neurotrophic. Neurological manifestations can range from nonspecific symptoms such as dizziness, headaches and olfactory disturbances to severe forms of neurological dysfunction. Some neurological complication can occur even after mild forms of respiratory disease. This study’s aims were to assess cerebrovascular reactivity in patients with nonspecific neurological symptoms after SARS-CoV-2 infection. A total of 25 patients, aged 33–62 years, who had nonspecific neurological symptoms after SARS-CoV-2 infection, as well as 25 healthy participants in the control group, were assessed for cerebrovascular reactivity according to transcranial color Doppler (TCCD) which we combined with a breath-holding test (BHT). In subjects after SARS-CoV-2 infection, there were statistically significantly lower flow velocities through the middle cerebral artery at rest period, lower maximum velocities at the end of the breath-holding period and lower breath holding index (BHI) in relation to the control group. Changes in cerebral artery flow rate velocities indicate poor cerebral vasoreactivity in the group after SARS-CoV-2 infection in regard to the control group and suggest vascular endothelial damage by the SARS-CoV-2 virus.

## 1. Introduction

Acute respiratory syndrome with coronavirus 2 (SARS-CoV-2) virus has been a major global health problem since 2020 year. As of March 2021, the SARS-CoV-2 pandemic has resulted in more than 125 million people worldwide infected and more than 2.8 million people have died (WHO COVID-19 Dashboard, March 2021). The SARS-CoV-2 virus has been known as a respiratory virus and the essential clinical feature is an acute respiratory infection [1], but from the beginning of the pandemic it has been clear that the virus is highly neurotrophic [2,3]. Patients with severe clinical manifestations of SARS-CoV-2 infection were more likely to experience neurological symptoms compared with those with mild disease [4]. The majority of these patients experienced prolonged headache, disturbance in consciousness, acute cerebrovascular disease (ischemic stroke, cerebral or subarachnoid hemorrhage), acute encephalopathy, encephalitis or meningitis, polyneuropathy, multiple sclerosis spectrum of disease and seizures. Milder forms of disease are often accompanied by nonspecific neurological complications such as headache, dizziness, ageusia (loss of taste), anosmia (loss of smell), myalgia and fatigue [5,6]. The SARS-CoV-2 virus membrane is characterized by the presence of the spike (S) glycoprotein which facilitates entry into neural, glial and endothelial cells which have angiotensin converting enzyme 2 (ACE2) receptors [7]. Several mechanisms may be involved in the pathophysiology of the virus as well as damage to the nervous system, but full mechanisms are still not fully understood. Neurological manifestations can be caused by non-specific complications of systemic infectious disease, inflammation of the nervous system or dysfunction of cerebral vasculature [8,9]. Vascular endothelial inflammation and SARS-CoV-2 complement-induced coagulopathy cause diffuse endothelial dysfunction, impaired vasoreactivity, in most severe cases associated with thrombus formation in the microcirculation [10,11]. As Hernadez-Fernandez et al. showed in their study, endothelial disruption is a basic mechanism of the virus pathophysiological process [12]. Hyperactivity of the host immune system and molecular mimicry may further aggravate brain damage and clinical picture [13] so as autoantibodies against heat shock proteins [14], prolonged hypoxia and electrolyte changes as a consequence of long-term respiratory disease also may contribute to the development of neurological complications. The involvement of the CNS may be related to poor prognosis and disease worsening, but even patients with mild respiratory symptoms can have some prolonged neurological symptoms. An increasing number of patients develop cerebrovascular disease after overcoming SARS-CoV-2 virus infection without having any risk factors prior to infection. Cerebrovascular disease is the second leading cause of death worldwide and some classic risk factors are well known, but new risk factors such as infectious agents have been documented recently [15]. Chronic infection, such as Chlamydia pneumonia, human cytomegalovirus, Helicobacter pylori, influenza virus, hepatitis C virus, etc., contribute to the development of cerebrovascular disease, causing changes in the small and large blood vessels of the brain [16]. Healthy brain arteries are capable of maintaining a constant cerebral blood supply via cerebral autoregulation mechanisms, despite changes in cerebral perfusion. A noninvasive, real-time, well tolerated and accurate method for the evaluation of main basal intracranial arteries, their flow rates and hemodynamic parameters is transcranial color Doppler (TCCD) [17,18] which provides useful information on peak systolic velocities (PSV), end diastolic velocities (EDV), mean velocities (MV), resistance index (RI) and pulsatility index (PI). In response to vasodilator stimulation such as CO2 inhalation, the breath-holding test (BHT) or acetazolamide administration, we can estimate cerebral vasoreactivity by measuring flow velocities in cerebral arteries and flow velocity changes induced by hypercarbia [19]. Impaired vasoreactivity and reduced reserve capacity of brain arteries are predisposing conditions for cerebrovascular disease [20]. The aim of our study was to assess cerebrovascular reactivity in patients after SARS-CoV-2 infection with nonspecific neurological symptoms, using TCCD and the breath-holding test. The hypothesis of our study is that patients after mild SARS-CoV-2 infection have impaired cerebral vasoreactivity.

## 2. Materials and Methods

### 2.1. Population Study

Analyzing the electronic database of the University Hospital Canter Split, we found 456 patients who sought help at a neurology clinic, polyclinic department, for nonspecific neurological symptoms such as headache, loss of sense of smell and taste, dizziness, and weakness from January to March 2021. Among all of them, we found 185 patients who, according to available database, had SARS-CoV-2 infection, and 72 of them who had it in the last 60 days. Patients were classified as mild, moderate or severe based on WHO Criteria [21] and only 66 of them met the criteria for mild forms of COVID-19 disease. Only 54 patients of these were without a significant risk factors for cerebrovascular disease. Among them, only 49 were considered to be suitable for our study because they were neither older than 62 years nor younger than 32 years. We excluded the younger age group from the study due to the possible increased elasticity of blood vessels in the brain that gives high flow rates, while we excluded the older age group due to increased resistance and increased vessel stiffness. Just 34 patients agreed to participate in our study and signed informed consent. Among them, only 28 patients had a good insonation window through the right temporal bone so that TCDP could collect data on the flow velocity through the middle cerebral artery. Three patients withdrew from the study for personal reasons. Within 2 weeks, subjects were contacted by phone. Before the collection of any data, all 25 remaining patients signed an individual informed consent form. Then, they were invited to attend a first interview when demographic data were collected, neurological examination performed, and somatic examination. Then, all subjects were measured by body weight and height, blood pressure, ECG and ultrasound of the blood vessels of the neck. A TCCD was then made according to the study protocol. We found a control group among healthcare professionals and post-graduate students. The basic condition was that they did not have SARS-CoV-2 infection, that they were negative on a real-time reverse PCR test and serological test for IgG and IgM antibodies to SARS-CoV-2 virus, and that they did not have significant risk factors for cerebrovascular disease. They also were measured for body weight, height, and blood pressure, and we recorded their ECG and ultrasound of the blood vessels of the neck, and TCCD as study protocol demanded.

### 2.2. Methods

We conducted a cross-sectional observational study. From each participant, written informed consent was obtained. The Ethics Committee of the University Hospital Split approved this study in March 2021 (class 500-03/21-01/39, NO 2181-147-01/06M.S-20-02). The test group was made up of 25 was patients who had mild respiratory symptoms of SARS-CoV-2 infection and a positive result of real-time reverse PCR test at the time of infection. They all had post-infection non-specific neurological symptoms such as smell and taste dysfunction, vertigo, headache, dizziness, myalgias or fatigue. All subjects overcame SARS-CoV infection from 28 to 50 days before TCCD recording. The control group was 25 healthy volunteers (postgraduate students or healthcare workers) who had no symptoms of infectious SARS-CoV-2 disease and who were negative for real-time reverse PCR test and seronegative for SARS-CoV IgG and IgM antibodies. All subjects were enrolled in March 2021. All participants were Caucasian adults, aged 33–62 years. Participant data include: age, gender, height, weight, body mass index, history of smoking, history of alcohol drinking, regular drugs use, amount of physical activity, hypertension, diabetes, hyperlipidemia, coronary heart disease, atrial fibrillation and prior cerebrovascular disease. The amount of physical activity was assessed using the International Physical Activity Questionnaire and are expressed as a minutes of moderate physical activity per week. None of the subjects from the SARS-CoV-2 group were hospitalized or treated on an outpatient basis for SARS-CoV-2 pneumonia, and they were treated exclusively with supportive therapy for SARS-CoV-2 infection (they did not use antibiotics, oxygen therapy, antiviral drugs or corticosteroids). We excluded all patients with a history of uncontrolled hypertension, unregulated diabetes mellitus, cerebrovascular disease, hematologic disease, atrial fibrillation, chronic heart disease or cancer, severe alcohol consumption (more than 10 alcoholic drinks per week), known occlusive disease of cerebral arteries, stenosis of the vertebral artery or external carotid artery more than 20%. We excluded all patients using anticoagulant or vasodilator drugs, hormone replacement therapy, β-blocking agents and calcium channel blockers (Table 1). The two groups of subjects were matched for age, gender, body mass index, systolic and diastolic blood pressure and amount of physical activity. All participants performed somatic and neurological status, electrocardiography testing, blood pressure testing, extra cranial carotid artery ultrasound, and transcranial Doppler ultrasonography with breath-holding test. We performed all ultrasonic measurements using the Hitachi Aloca Arietta 70 Ultrasound system (2.0 MHz frequency transducer). All tests were performed after working hours, in a quiet and peaceful room, possible sources of sound and light interference were excluded, and the patient was lying in supine position. Transcranial Doppler ultrasonography was performed first in the resting phase which lasted 5 min, after which, participants hold their breath as long as they could. After the breath-holding period, the subject would breathe normally for 5 min. We repeated the procedure three times for of each participant. Blood flow signals were detected using a 2.0 MHz pulse probe at a depth of 52–64 mm via right temporal bone window. We insonated arteries with special focus on the right middle cerebral artery (MCA). Measurements in our study included peak systolic velocity (PSV), end diastolic velocity (EDV) and mean velocity (MV), resistance index (RI) and pulsatility index (PI) of right middle cerebral artery (MCA). We measured PSV, EDV, MV, RI and PI values on right MCA continuously during the rest and after the breath-holding period. We particularly focused on velocities at beginning of respiratory arrest and at the very end of the respiratory arrest period. We recorded the velocities at rest as PSV rest, EDV rest, MV rest, RI rest and PI rest and at the end of breath-holding period as PSV max, EDV max, MV max, RI max and PI max. Furthermore, we also measured the length of time for each subject to stop breathing (time of breath-holding—BHT). We determined the mean values of each variable in all three measurements. Vascular reactivity was determined upon calculating the breath holding index (BHI) as the percent of velocity increase from resting baseline values (PSVmax/PSVrest) divided by breath holding time (BHT).

### 2.3. Statistical Analysis

Statistical significance was set to *p* < 0.05 and all confidence intervals were given at the 95% level. For numeric variables Shapiro–Wilk test was used to indicate deviation from normal distribution. Numeric variables were presented by median (Q1–Q3) or by mean ± SD. Statistical significance of the differences of categorical variables was calculated by the chi-square test. Analysis of differences of numeric variables between two groups was carried out by the independent-samples T-test or by the Mann–Whitney U test. Analysis of differences between two measurements of numeric variables was carried out by paired-samples T-test. Statistical analysis was carried out by SPSS 20.

## 3. Results

### 3.1. Patient Characteristics and Measured Variables 

Our study included 25 subjects who had nonspecific neurological symptoms after SARS-CoV-2 infection and 25 subjects in the control group. For each subject, we measured blood flow velocities through the midbrain artery: peak systolic velocity (PSV), end diastolic velocity (EDV), mean velocity (MV), and resistance indices (RI) and pulsation rate (PI). Velocities were measured at rest (PSVrest, EDVrest, MVrest, RIrest, PIrest) and three times after the breath-holding test (BHT) as PSVmax, EDVmax, MVmax, RImax, PImax. All examined quantitative variables (blood flow velocities through the middle cerebral artery) had a normal distribution according to the Kolmogorov–Smirnov test (*p* > 0.05), both in the group of subjects after SARS-CoV-2 infection and in the control group. There were no clinical or statistically significant differences (*p* > 0.05) between three repeated measurements after the breath-holding test (BHT) in the group of subjects after SARS-CoV-2 infection and in the control group, so we made an arithmetic mean of three measurements after the breath-holding test (BHT) for each subject and we used it in further analysis. Groups were aligned by gender (χ^2^ = 0.089; *p* = 0.765), age (T = 0.388; *p* = 0.699), body mass index—BMI (T = 0.717; *p* = 0.477), systolic arterial pressure (T = 1.7; *p* = 0.099), diastolic arterial pressure (T = 1.5; *p*= 0.129) and physical activity (T = 0.472, *p* = 0.639).

There were only seven diabetics in both groups. Out of the total number, there were only three hypertensive subjects. One participant had elevated blood lipids. One subject from each group drank more than 10 alcoholic beverages per week. In both groups, a total of seven subjects regularly smoked cigars (Table 2 shows demographic data and some of clinical parameters).

### 3.2. Disease Symptoms in a Group of Subjects after SARS-CoV-2 Infection

In the group of subjects after SARS-CoV-2 infection, according to nonspecific neurological symptoms, anosmic symptoms had 14 (56%) subjects, dysgeusia was present in 14 (56%) subjects, 10 (40%) subjects had dizziness, 17 (68%) of them had headaches, 18 (72%) had fatigue and myalgia was present in 14 (56%) of participants. None of the subjects had symptoms of stroke, epileptic seizures, signs of meningoencephalitis, narrowing of consciousness movement disorders, demyelinating disease or acute polyradiculoneuritis. According to the general symptoms of the disease, all subjects had fever (25, 100%), (80%) 20 of them had a cough, 10 subjects had sore throats (40%), 2 subjects had gastrointestinal symptoms (8%) and 4 (16%) had a rash. None of the subjects had dyspnea. None of the subjects were hospitalized for symptoms associated with SARS-CoV-2 virus infection.

### 3.3. Comparison of Flow Velocities through Middle Cerebral Artery at Rest and after Breath-holding Test between Test Groups

Table 3 shows the values of flow velocities through the middle cerebral artery in relation to the groups of subjects.

### 3.4. Comparison of Velocities in Middle Cerebral Artery at Rest between Group of Subjects after SARS-CoV-2 Infection and Control Group 

At the rest period, subjects after SARS-CoV-2 infection had statistically significantly lower all measured velocities parameters compared to control group except PI:

-lower PSV (T = 4.5; *p* < 0.001), arithmetic mean difference was 12.5 (95%CI; 6.8 to 18).

-lower EDV (T = 3.2; *p* = 0.002), arithmetic mean difference was 4.6 (95%CI; 1.7 to 7.5).

-lower MV (T = 4.4; *p* < 0.001), arithmetic mean difference was 9.2 (95%CI; 5 to 13.5).

-lower RI (T = 3.1; *p* = 0.003), arithmetic mean difference was 0.03 (95%CI 0.01 do 0.05).

We did not prove statistically significant difference for PI values (T = 0.856; *p* = 0.396). (Table 4).

### 3.5. Comparison of Flow Velocities through Middle Cerebral Artery after a Breath-Holding Test between Group of Subjects after SARS-CoV-2 Infection and a Control Group

After the breath-holding test, velocity parameters were higher than at the rest period for both groups, but the group of subjects after SARS-CoV-2 infection had statistically significantly lower all measured velocity parameters compared to the control group, except PI:

-lower PSV max (T = 14.3; 0 < 0.001), arithmetic mean difference was 39.4 (95%CI; 34 to 45).

-lower EDV max (T = 9.1; *p* < 0.001), arithmetic mean difference was 12 (95%CI; 9 to 14.6)

-lower MV max (T = 7.7; *p* < 0.001), arithmetic mean difference was 15.4 (95%CI; 11 to 19).

-lower RI max (T = 3.6; *p* = 0.001), arithmetic mean difference was 0.015 (95%CI; 0.006 to 0.023).

After the breath-holding test, there was no statistically significant difference of PI max value between the two groups (T = 1.05; *p* < 0.300) (Table 5).

The mean value of breath holding time in both groups was 37 ± 4 s (minimum 29 to maximum 47); there was no statistically significant difference between the two groups of subjects (T = 0.951; *p* = 0.346) (Table 5). 

### 3.6. Comparison of Changes in Flow Velocities through Middle Cerebral Artery after Breath-Holding Test and Rest Period between Two Examined Groups and Breath Holding Index (BHI)

#### Mann–Whitney U Test

After the breath-holding test, relative increases in flow velocities in the control group were statistically significantly greater than in the group of subjects after SARS-CoV-2 infection for PSV (2.4 times higher, median difference was 20 (95% CI: 15.4–23.04) (z = 6.01; *p* < 0.001) and EDV (1.2 times higher, median differences was 6 (95% CI: −0.38 to 13.54) (z = 2.6; *p* = 0.010). From these two parameters, we calculated the breath holding index, and that index was statistically significantly higher in control subjects compared to subjects who had SARS-CoV-2 infection. The median difference was 0.55 (95%CI; 0.41–0.69) (z = 6; *p* < 0.001).

### 3.7. Correlation of Flow Velocities through Middle Cerebral Artery with Age of the Subjects in Each Group

We did not prove a statistically significant correlation between examined flow velocities through the middle cerebral artery in relation to age of the subjects in both examined groups. Pearson’s correlation coefficient for age was for RI r = −0.031, for PI, the correlation coefficient was r = −0.075, for PSV, the correlation coefficient was r = −0.048, for EDV, the correlation coefficient was r = 0.115, and for MV, the correlation coefficient was r = −0.252.

### 3.8. Correlation of Changes in Flow Velocities Parameters through Middle Cerebral Artery in Relation to Time from Onset of Symptoms

The median time from onset of symptoms of SARS-CoV-2 virus infection and TCCD measurements was 36 days (minimum–maximum: 28 to 50 days). We did not prove a statistically significant association of flow velocities parameters through the middle cerebral artery with time from onset of SARS-CoV-2 virus infection. All Spearman correlation coefficients were less than 0.4, *p* > 0.05.

### 3.9. Correlation of Flow Velocities through Middle Cerebral Artery with Gender of the Subjects after SARS-CoV-2 Infection

We did not prove a statistically significant correlation between the examined flow velocities through the middle cerebral artery in the rest period (PSV; t = 1.19, *p* = 0.244, EDV; t = 0.082, *p* = 0.935, MV; t = 0.427, *p* = 0.673, RI; t = 0.154, *p* = 0.879, PI; t = 0.228, *p* = 0.822) and after breath-holding test (PSV; t = 1.151, *p* = 0.260, EDV; t = 0.104, *p* = 0.918, MV; t = 0.379, *p* = 0.708, RI: t = 0.347, *p* = 0.732, PI; t = 0.628, *p* = 0.536) in relation to the gender of the subjects in SARS-CoV-2 group.

## 4. Discussion

To date, there has been no study investigating cerebral vasoreactivity in patients after SARS-CoV-2 infection. In our study, we showed that patients even after mild SARS-CoV-2 infection have impaired cerebral vasoreactivity. Values of flow velocities through the middle cerebral artery at the rest period and after breath-holding test were statistically significantly lower in subjects who had SARS-CoV-2 infection than in the control group. A smaller relative increase in PSV and EDV values in the group of subjects with SARS-CoV-2 infection leads to a consequent lower breath holding index (BHI) which directly indicates impaired vasoreactivity and weaker vasoconstriction response to hypercarbia in patients after SARS-CoV-2 infection. We did not prove a statistically significant correlation between examined flow velocities through the middle cerebral artery in relation to age, gender or with time from the onset of SARS-CoV-2 virus infection symptoms.

Romero-Sanchez et al. in their study showed that neurologic manifestations are very common in hospitalized patients with SARS-CoV-2 infection and more than half of them (57.4%) developed some form of neurologic symptom [22]. Nonspecific symptoms were present mostly in the early stages and in less severe cases, and anosmia and dysgeusia were common as a first clinical manifestation of SARS-CoV-2 disease. Serious neurologic complications were less frequent but can cause death in about 4.1% patients. In our study, we recruited subjects from the outpatient clinic’s database so there were no serious neurological complications, no one was hospitalized, and due to mild and nonspecific neurological symptoms, examination was one month after the onset of infection. According to the data from this study, the most common nonspecific neurological symptoms in our study were dysgeusia (56%), anosmia (56%), headache (68%) and fatigue (72%).

Hernandez-Fernandez et al. showed in their study [12] that endothelial disruption is the primary mechanism of damage in SARS-CoV-2 patients who had cerebeovascular disease related to infection. Thrombotic microangiopathy, loss of vasoreactivity, increased bleeding predisposition and increased hypercoagulability along with systemic complications were the cause for poor clinical prognosis of these patients. SARS-CoV-2 patients who had cerebrovascular disease had an unfavorable clinical outcome (73.9% of them had modified Rankin score 4–6), and a high mortality rate (34.8%). Age was the only independent predictive factor of poor prognosis and it was a high incidence of large vessel occlusion (58.8%), and unexpectedly, many strokes were in the vertebrobasilar location (35.3%). As our subjects had mild symptoms of SARS-CoV-2 infection, both general and neurological, cerebral vasoreactivity disorder is an expression of SARS-CoV-2 virus neurotropicity and impaired endothelial function.

Chen et al. studied the frequency of neurological symptoms and complications in SARS-CoV-2 patients [23], and concluded that headache, dizziness, taste and smell dysfunctions were the most frequently reported neurological symptoms with an overall frequency of greater than 4% of the populations studied. In our study, it was not possible to determine the real prevalence of mild neurological symptoms because the database was outpatient clinic-based and did not necessarily reflect the incidence of neurologic complications of patients with SARS-CoV-2 in the community. However, the most common symptoms in the Chen et al. study were the most common also in our subjects.

The basic task of the cerebral arteries is to ensure a sufficient supply of nutrients and energy for the brain. The anatomical and functional organization of cerebral blood flow allows brain perfusion to be stable even in conditions of increased energy demand. The Willis circuit allows an anatomical and small vessel network system allows a functional aspect of physiological reserve. The basic principle of cerebral vasoreactivity is the dilatation of small blood vessels, which increase flow velocities through large basal arteries [24]. TCCD is a useful tool for the assessment of flow velocities in the large brain arteries [25] and to indirectly estimate cerebrovascular reserve capacity, as Widder showed in his book [26]. Flow velocities through large cerebral arteries increase after the breath-holding test, which induces hypercarbia and hypoxia [27]. The middle cerebral artery is suitable for examining these parameters, because most of the flow through the cerebral arteries goes through that large blood vessel. 

SARS-CoV-2 virus binds to angiotensin converting enzyme 2 receptors for entry into cells, a receptor which can be found in numerous brain cells: neurons, glial cells, vascular endothelium and smooth muscle [28]. For example, in their study, Al-Ramadan et al. found that SARs-CoV-2 virus can cause both acute and long-term neurological complications in many patients [29]. Cerebrovascular disease in SARS-CoV-2 infection, according to Al-Ramadan et al., might be due to SARS-CoV-2 dysregulation of the renin–angiotensin system (RAS) by acting on ACE2 receptors, direct infection of the endothelial cell which can results in endothelial dysfunction and impaired cerebral vasoreactivity. Like other virus infections, the SARS-CoV-2 virus can cause severe endothelial dysfunction. Pavicic Ivelja et al. found that chronic hepatitis C patients have altered cerebrovascular reactivity and these negative effects on cerebrovascular hemodynamics could contribute to the increased risk of cerebrovascular disease [30]. Chow et al. found similar changes in patients with HIV infection and their cerebrovascular endothelial dysfunction may independently contribute to cognitive impairment [31]. Our study of SARS-CoV-2 subjects clearly showed impaired cerebrovascular reactivity, which had negative effects on cerebrovascular hemodynamics and can increase the risk of cerebrovascular disease. It is significant that subjects from the SARS-CoV-2 group did not have significant risk factors for cerebrovascular disease. Impaired vasoreactivity, and a weaker response to hypercarbia after the breath-holding test in patients after SARS-CoV-2 infection may lead to decreased vascular capacity and vascular reserve, especially in conditions of increased demand [32,33]. 

Transcranial color Doppler is a non-invasive, cheap and reproducible method, and it has been used for a long time in clinical practice for monitoring for cerebral vasospasm following subarachnoid hemorrhage, evaluation occlusive cerebrovascular disease and the detection of cerebral microembolic signals. It also can be used to identify patients with exhausted cerebrovascular reserve and for the assessment of cerebral autoregulation, as we showed in our study. In previous studies, TCCD has been shown to be a good method for assessing brain vasoreactivity in studies for patients with cerebrovascular disease, like Silvestrini et al. found in their study [33]. They concluded that alterations in cerebral hemodynamic function may play a relevant role in the occurrence of stroke in patients with carotid artery disease. Silvestrini et al. also concluded that the reduction in the BHI values strongly increases the probability of occurrence of a cerebrovascular ischemic event. All these studies have shown that the reduction in vasodilatatory capacity leads to the reduction in vascular adaptability and can be a precursor for the development of cerebrovascular disease [34,35,36]. Similar studies have not yet been published with SARS-CoV-2 patients and this is the first such study to occur during this pandemic. TCCD with the addition of the breath-holding test brings added value for these patients because induced hypercarbia increases the susceptibility to vasoreactivity disorder. There are no biological markers to accurately assess brain vasoreactivity and TCCD is a reliable method for assessing that. These patients, in the future, can be severely affected due to the inability to respond to conditions of increased cerebral perfusion demand, due to a lack of vascular reserve [37,38]. These patients are at increased risk of developing stroke [5,39], but also at increased risk that the ischemic zone is bigger than in patients with preserved vascular reserve [40,41]. Cerebral vasoreactivity can also be impaired in neurodegenerative diseases. Urbanova et al. in their study showed [42] that decreased cerebrovascular reserve capacity and altered vasoreactivity can be found in patients with Alzheimer’s disease as a sign of microangiopathy even without severe underlying atherosclerosis and it can be identified using TCCD along with the breath-holding test (BHI). Shim et al. found in their study that underlying microangiopathy can be a mechanism in Alzheimer’s disease patients and showed there was an association between the impaired function of cerebral microvessels and cognitive impairment [43]. Espino-Ojeda et al. in their study found that Parkinson’s disease patients are prone to exhibit diminished cerebrovascular reserve and altered cerebral vasoreactivity in comparison with healthy individuals [44]. In the future, we need additional vasoreactivity studies, we need to monitor patients with impaired vasoreactivity, and we need rigorous and systematic longitudinal follow up. Time is our ally, and future studies should be performed on a larger number of patients if we want to prevent the possible numerous neurological complications of this pandemic. 

The main limitation to this study is the small number of subjects included in both groups. The SARS-CoV-2 virus pandemic has significantly changed the work of the overall health care system, and also the neurological outpatient clinic, and there are certainly more patients who have nonspecific neurological symptoms and who need to be examined by a neurologist, but they were not registered in the hospital system at the time we recruited our subjects. Additionally, a short period of time had passed since the onset of the SARS-CoV-2 symptoms in our subjects, so the long-term consequences of impaired vasoreactivity have yet to be investigated through systematic longitudinal follow-up. One of the limiting factors of the study is TCCD itself as a method of assessing brain vasoreactivity, because the quality of the findings largely depends on the quality of the temporal bone window through which the middle cerebral artery is insonated. The hemodynamic effect of breath holding is lower than that of carbon dioxide inhalation or acetazolamide injection. All TCCD testing in our study was carried out by one examiner who has 15 years of experience working with TCCD, and thus we avoided a possible interpersonal difference depending on the experience of the examiner.

## 5. Conclusions

Neurological manifestations prior, during and after SARS-CoV-2 virus infection are increasingly diagnosed but pathophysiologically are not fully understood. The physicians must consider nonspecific neurological symptoms as a clear sign of virus neurotropism. The long-term effect of the neuroinvasive potential of the SARS-CoV-2 virus may increase the risk of cerebrovascular disease. In this study, we have showed that patients after SARS-CoV-2 virus infection had significantly lower average peak systolic, end-diastolic and mean velocity values at the end of the breath-holding procedure. Additionally, the breath holding index was significantly lower in the SARS-CoV-2 group than in the healthy control group. It is a direct expression of impaired cerebral artery vasoreactivity in patients with neurological symptoms after overcoming mild respiratory SARS-CoV-2 infection. Cerebral vasoreactivity disorder points to a damaged mechanism of brain vasoregulation and indicates patients who without other risk factors will be predisposed for cerebrovascular disease. Such patients should be identified, properly selected and treated to reduce possible major health problems. Additional studies are needed to determine the association of neurological symptoms after SARS-CoV-2 infection and changes in cerebral artery vasoreactivity, as well as the impact of time on these changes.

## Figures and Tables

**Table 1:** Inclusion and exclusion criteria.

Inclusion Criteria	Exclusion Criteria
Age from 30 to 65 years	Age under 30 and over 65 years
Mild form of respiratory SARS-CoV-2 disease	Severe or critical form of SARS-CoV-2 pulmonary infection
Non-specific neurological symptoms such as smell and taste dysfunction, vertigo, headache, dizziness or fatigue	Disturbance in consciousness, acute cerebrovascular disease (ischemic stroke, cerebral hemorrhage, subarachnoid hemorrhage), acute encephalopathy, encephalitis or meningitis, polyneuropathy, demyelinating spectrum of disease and seizures.
SARS-CoV infection from 30 to 60 days before TCCD recording	More than 60 days from infection start and TCCD recording
Treated exclusively with supportive therapy	Use of antibiotics, corticosteroids, oxygen for SARS-CoV-2 infection
Diagnosis confirmed by a positive result of real-time reverse PCR test by nasal/pharyngeal swabs	History of uncontrolled hypertension, nonregulated diabetes mellitus, cerebrovascular disease, hematologic disease, atrial fibrillation, chronic heart disease or cancer
	Severe alcohol consumption (more than 10 drinks per week)
	Stenosis of extracranial vertebrobasilar artery > 20%
	Stenosis of extracranial carotid artery > 20%.
For control group negative real time reverese PCR test by nasal/pharyngeal swabs Negative serological IgM and IgG test on SARS-CoV-2 virus	Known occlusive disease of intracranial cerebral arteries
No SARS-CoV-2 symptoms at all	Using anticoagulant drug, vasodilatory drugs, hormone replacement therapy, β-blocking agents, calcium channel blockers

**Table 2:** Display of the number (%) of subjects according to qualitative data and arithmetic mean ± SD and quantitative data in relation to the examined groups (subjects after SARS-CoV-2 infection and subjects of the control group).

		Subject Groups		
		after SARS-CoV-2 Infection	Control Group	*p*
gender	Male	16 (64)	17 (68)	0.765 *
	Female	9 (36)	8 (32)	
diabetes		4 (16)	3 (12)	
hypertension		3 (12)	1 (4)	
hyperlipidemia		1 (4)	0 (0)	
alcohol		1 (4)	1 (4)	
smoking		4 (16)	3 (12)	
age (years)		46.6 ± 8.5	45.7 ± 7.4	0.699 **
BMI (kg/m^2^)		25.6 ± 2.9	25 ± 2.8	0.477 **
RR systolic (mmHg)		126.7 ± 11.6	120.8 ± 13.1	0.099 **
RR diastolic (mmHg)		78.4 ± 9.6	74.6 ± 7.9	0.129 **
Physical activity (minutes per week of moderate activity)		179.3 ± 25	182 ± 15	0.639 **

* χ^2^ test; ** T test.

**Table 3:** Representation of arithmetic means of flow velocities through middle cerebral artery and standard deviations (±SD) (95% CI) of these parameters.

	Subject Groups		
	after SARS-CoV-2 Infection	Controls	*p* *
Subjects at rest			
PSV (cm/s)	107 ± 12.7 (102–112)	120 ± 5.5 (117–122)	<0.001
EDV (cm/s)	51.9 ± 4.6 (50–54)	56.5 ± 5.5 (54–59)	0.002
MV (cm/s)	72.1 ± 7.3 (69–75)	81.3 ± 7.5 (78–84)	<0.001
RI	0.53 ± 0.02 (0.50–0.53)	0.55 ± 0.04 (0.53–0.57)	0.003
PI	0.77 ± 0.07 (0.74–0.79)	0.78 ± 0.05 (0.78–0.76)	0.396
Subjects after breath-holding test			
PSV (cm/s)	122 ± 11.3 (117–127)	162 ± 7.8 (158–165)	<0.001
EDV (cm/s)	69.3 ± 3.5 (68–71)	81 ± 5.5 (79–84)	<0.001
MV (cm/s)	94.7 ± 8.6 (91–98)	110.1 ± 5 (108–112)	<0.001
RI	0.53 ± 0.02 (0.52–0.54)	0.51 ± 0.01 (0.51.0.52)	<0.001
PI	0.78 ± 0.08 (0.74–0.81)	0.76 ± 0.04 (0.74–0.77)	0.300

Legend: PSV—peak systolic velocity, EDV—end-diastolic velocity, MV—mean flow velocity, RI—resistance index, PI—pulsatility index, T test for independent samples. * T test for independent samples.

**Table 4:** The frequency of nonspecific neurological and general symptoms in subjects who had mild form of SARS-CoV-2 infection.

	Symptoms in SARS-CoV-2 Group	
Neurological Symptoms	SARS-CoV-2	Control
	*n*	*n*
Anosmia	14 (56)	0 (0)
Dysgeusia	14 (56)	0 (0)
Dizziness	10 (40)	0 (0)
Headache	17 (68)	0 (0)
Fatigue	18 (72)	0 (0)
Myalgia	14 (56)	0 (0)
Symptoms of infective disease		
Fever	25 (100)	0 (0)
Cough	20 (80)	0 (0)
Sore throat	10 (40)	0 (0)
Gastrointestinal symptoms	2 (24)	0 (0)
Rash	4 (8)	0 (0)
Dyspnea	0 (0)	0 (0)

**Table 5:** Median (Q1–Q3) presentation of changes in flow velocities rates through middle cerebral artery after breath-holding test and rest period in relation to groups of subjects.

	Subjects Groups		
	after SARS-CoV-2 Infection	Controls	*p*
Relative change of velocities parameters after breath-holding test compared to values at resting period (%)			
∆PSV (%)	14 (11–19)	34 (32–39)	<0.001
∆EDV (%)	36 (30–38)	42 (35–50)	0.010
∆MV (%)	31 (26–38)	36 (25–41)	0.222
∆RI (%)	1.4 (−0.6 to 3.9)	−5 (−12 to −2)	<0.001
∆PI (%)	2.3 (0–3.3)	−4 (−6 to 1.4)	0.013
Breath holding index (BHI)	0.426 (0.28–0.57)	0.98 (0.81–1.12)	<0.001

**Legend:** PSV—peak systolic velocity, EDV—end-diastolic velocity, MV—mean flow velocity, RI—resistance index, PI—pulsatility index.
